# Liquid biopsy for disease monitoring after anti‐CD19 chimeric antigen receptor T cell in diffuse large B‐cell lymphoma

**DOI:** 10.1002/jha2.131

**Published:** 2020-12-09

**Authors:** Clara Maluquer, Beatriz Bellosillo, Alberto Mussetti, Eva Domingo‐Domènech, Rocío Parody, Lierni Fernández‐Ibarrondo, Roser Velasco, Gabriel Moreno‐González, Gabriela Sanz, Montserrat Cortés, Anna Sureda

**Affiliations:** ^1^ Department of Hematology Catalan Institute of Oncology‐Hospitalet, IDIBELL (Institut d'Investigació Biomèdica de Bellvitge), University of Barcelona Barcelona Spain; ^2^ Pathology Department Hospital del Mar. IMIM (Hospital del Mar Medical Research Institute) Barcelona Spain; ^3^ Unit of Neuro‐Oncology Department of Neurology University Hospital of Bellvitge Catalan Institute of Oncology IDIBELL, University of Barcelona Barcelona Spain; ^4^ Intensive Care Unit Catalan Institute of Oncology University Hospital of Bellvitge IDIBELL, University of Barcelona Barcelona Spain; ^5^ Radiodiagnostic Unit, IDI (Institut de diagnòstic per la imatge) University of Bellvitge Barcelona Spain

**Keywords:** CART therapy, liquid biopsy, relapsed/refractory diffuse large B‐cell lymphoma

## Abstract

**Objectives:**

Chimeric antigen receptor T cells (CARTs) against CD19 antigen represent an effective therapy for relapsed/refractory diffuse large B‐cell lymphoma (rrDLBCL). There is no diagnostic test able to predict which patients with residual disease will relapse from those that will reach a delayed complete response. Positron emission tomography/computed tomography scan (PET‐CT) is characterized by a significant number of false positive results after immunotherapy. Circulating tumor DNA (ctDNA) may be a good‐useful tool to quantify minimal residual disease and for monitoring disease response.

**Methods:**

We present a patient with DLBCL treated with CART cells in which we tested the combined use of ctDNA and PET‐CT scan.

**Results:**

Disease reassessment with PET‐CT scan showed a partial remission (3 weeks) and a very good partial remission (2 months). A clinical progression at 3 months was confirmed with PET‐CT scan. Levels of ctDNA progressively decreased and became undetectable. An initial increase in KMT2D p.E4385G variant allele frequency confirmed disease progression.

**Conclusions:**

Our case shows how the complementary use of ctDNA and PET‐CT scan could be a helpful tool in the clinical management of these patients.

Chimeric antigen receptor T cells (CARTs) against CD19 antigen represent an effective therapy against refractory diffuse large B‐cell lymphoma (DLBCL). Patients who do not reach complete remission at first evaluation after infusion will probably relapse [1]. Positron emission tomography scan (PET‐CT) scan is the standard tool for disease monitoring but patients treated with immunotherapy approaches may present a high rate of false positive results due to posttreatment inflammatory changes [2]. This might also happen after CARTs infusion. The combination of circulating tumoral DNA (ctDNA) detection and PET‐CT scan has demonstrated to be valid as disease monitoring strategy in patients with DLBCL. Detection of molecular disease in plasma may precede the demonstration of disease relapse by PET‐CT and may have an improved specificity and a similar sensitivity with respect to PET‐CT scan alone [3]. There is an unmet clinical need in recognizing which patients are at increased risk of relapse after CARTs infusion. Liquid biopsy could be a key element in this setting; ctDNA is a quantitative test not influenced by inflammatory artifacts and it has already demonstrated prognostic significance in monitoring CARTs response in DLBCL patients [4, 5]. Herein, we present a high‐risk patient with DLBCL treated with CART cells, in which we tested the combined use of ctDNA and PET‐CT scan after infusion.

A 48‐year‐old male was diagnosed of stage IVB IPI of three DLBCL in August 2018 because of a clinical history of generalized lymphoadenopaties and constitutional syndrome. Inguinal lymph node biopsy showed a dense infiltrate with large lymphocytes that expressed CD20 and BCL6. CMYC, MUM1, Ki‐67, and P53 immunostainings were positive. A PET‐CT scan showed stage IV disease. The disease was refractory to three lines of chemoimmunotherapy (R‐CHOP, R‐GDP, and R‐ESHAP) and the patient was considered candidate to receive anti‐CD19 CARTs therapy. Tisagenlecleucel was infused on July 22, 2019 after lymphodepleting therapy with fludarabine 25 mg/m^2^ and cyclophosphamide 250 mg/m^2^ for 3 days. After infusion, he developed cytokine release syndrome grade 3 (ASTCT criteria) [6] and associated neurotoxicity grade 1 at day +4 treated with tocilizumab, hemophagocytic syndrome at day +6 treated with high‐dose dexamethasone, and was finally discharged on day +45. ctDNA samples were collected at day −8, +2, +12, +42, and +52 and at disease relapse (day +92). PET‐CT scans were performed at day −7, +17, +47, and +91 after infusion. To track ctDNA in the liquid biopsy samples, we first performed tissue genotyping with next‐generation sequencing from the lymph node biopsy that was performed after the first‐line therapy. Libraries were prepared using a custom panel that covered the whole codifying region of 17 genes involved in lymphomagenesis and progression/transformation (QIAseq Custom DNA Panels, Qiagen): EZH2, KMT2D, CREBBP, TNFRS14, STAT6, FAS, MYC, TP53, PIM, B2MG, CD58, BCL2, CARD11, CD79B, MYD88, TNFAIP3, and FOXO1. Molecular profiling of the tissue showed the coexistence of five mutations in five genes (Figure 1). CARD11 mutation had an allele frequency close to 50% that suggested it might be of germ line origin, and therefore, KMT2D p.Glu4385Gly was the tumor‐specific mutation chosen for patient follow‐up. We designed an allele‐specific custom TaqMan assay for the KMT2D mutation and assessed all the available plasma samples. ctDNA was obtained manually from 2 mL of plasma (MagMAX Cell‐Free DNA Isolation Kit; Thermo Fisher Scientific, South San Francisco, CA), quantified by Qubit (Thermo Fisher Scientific) with the Qubit High Sensitivity assay kit, and assessed by digital PCR in a QuantStudio 3D digital PCR system with the QuantStudio 3D AnalysisSuite Cloud Software (Thermo Fisher Scientific). Disease reassessment with PET‐CT scan showed a partial remission at +3 weeks and a very good partial remission at +2 months. Unfortunately, after 3 months he presented clinical progression confirmed with PET‐CT scan. The percentage of KMT2D p.Glu4385Gly mutation detected in plasma during the treatment is shown in Figure 1. There was an initial reduction of ctDNA eventually related to the tumor lysis due to lymphodepletion followed by a significant increase potentially correlated with cellular lysis due to CARTs‐related cytotoxic effect during the first week after infusion. Levels of ctDNA (day +42 and day +52) progressively decreased and became undetectable. These values correlated the + 3 weeks and + 2 months PET‐CT scans results. At +3 months, PET‐CT scan showed disease relapse. Standard biopsy was not feasible. A liquid biopsy was performed; an initial increase in KMT2D p.E4385G variant allele frequency confirmed the progression of disease. Due to the lack of effective salvage therapies, palliative care was started. The patient died 4 months after CARTs infusion.

**FIGURE 1:**
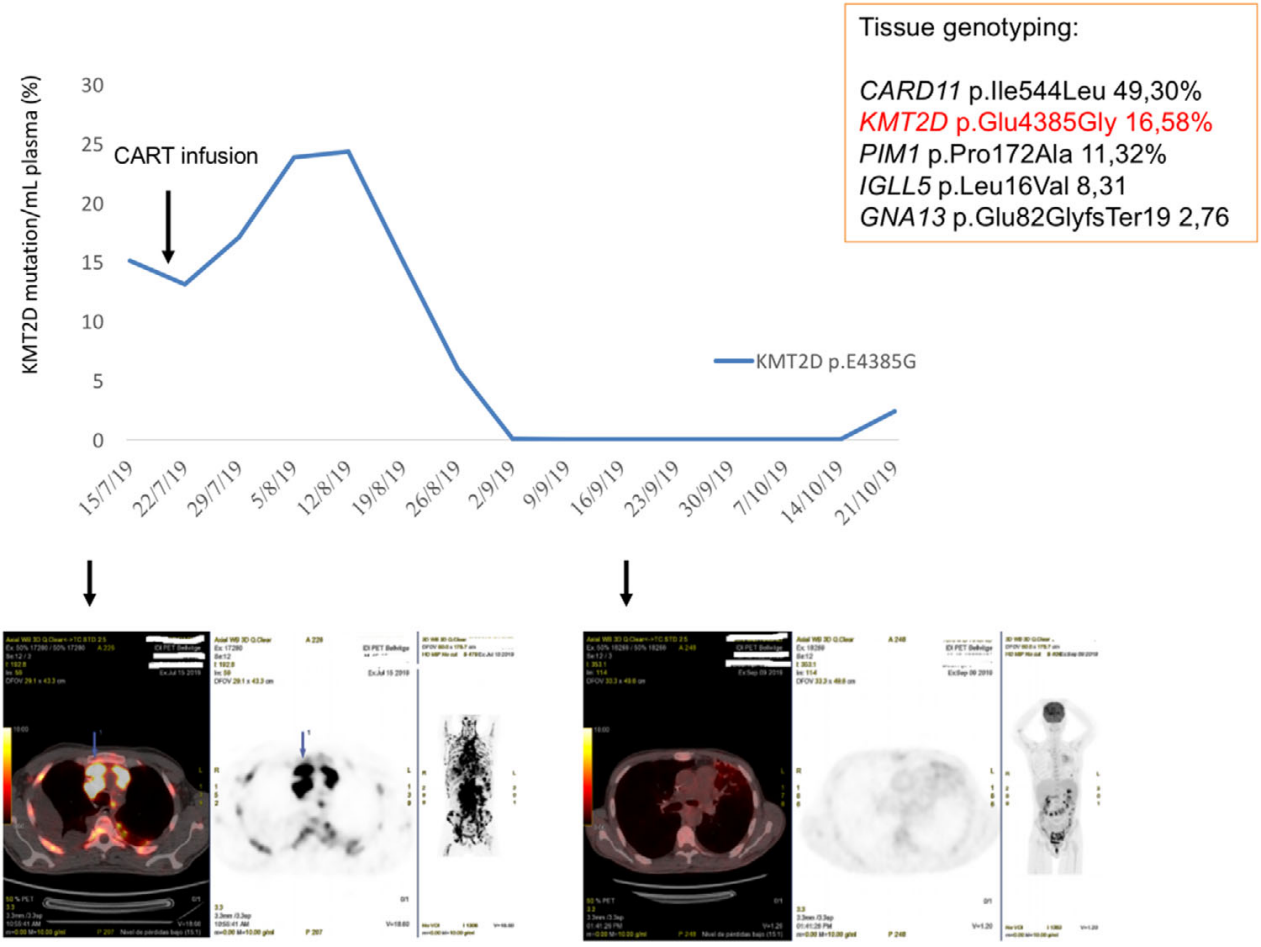
ctDNA monitoring since CART infusion until relapse. PET‐CT monitoring before CART infusion and 2 months after

The use of ctDNA as tumor monitoring strategy in association with radiological methods might be of potential scientific and clinical interest in this setting. Relapse post‐CARTs are associated with a very short survival. Having a test able to discriminate between patients at high or low‐risk of relapse could help the physician to decide which patients could benefit from consolidation strategies. The use of ctDNA for DLBCL monitoring after CARTs therapy still requires validation on large prospective cohorts of patients. In our case, ctDNA supported the results of standard PET‐CT scan for monitoring purposes; this is of paramount importance considering the radiological artifacts related to immunotherapies. Second, liquid biopsy avoided to expose the patient to high‐risk surgical intervention to confirm disease relapse. Thanks to the readily available results of ctDNA, we were able to take the important clinical decision of palliative care initiation. Unfortunately, in this case ctDNA was not able to predict disease relapse. Digital PCR was selected as the technique of choice to increase analytical sensitivity; however, a broader molecular profiling using NGS‐based assays such as CAPP‐SEQ could improve detection of resistance mechanisms. Nevertheless, the combined use of clinical, genetic, and radiological characteristics of the lymphoma should be used to assess the risk of relapse. Finally, ctDNA was able to describe a rapid decrease in disease burden at +2 months from CARTs infusion, suggesting the strength of the beneficial effect of CART cells in this highly refractory group of patients. Taking into consideration that disease response after CARTs is evaluated at 1 and 3 months after infusion by means of PET‐CT scan, the addition of complementary strategies to analyze disease evolution would help to clarify the kinetics of such a therapy. A better understanding could help to anticipate consolidation strategies. In conclusion, our case showed how the complementary use of ctDNA post‐CARTs therapy in DLBCL could be a helpful tool in the clinical management of these patients. Larger studies and a standardization of ctDNA techniques are required before the incorporation of this test into the clinical practice.

## CONFLICT OF INTEREST

AS and AM have received speakers’ fees from Novartis; the rest of the co‐authors declare no conflict of interest.

*We thank CERCA Programme / Generalitat de Catalunya for institutional support*.
